# Effect of *Launaea procumbens* on thyroid glands lipid peroxidation and hormonal dysfunction: a randomized control trial

**DOI:** 10.1186/s12944-017-0557-8

**Published:** 2017-09-11

**Authors:** Rahmat Ali Khan

**Affiliations:** Department of Biotechnology, Faculty of Biological Sciences, University of Science and Technology, Bannu, KPK Pakistan

**Keywords:** *Launaea Procumbens*, Oxidative stress, Antioxidant enzymes, T_3_, T_4_

## Abstract

**Background:**

*Launaea procumbens* (Roxb.) Amin is traditionally used in Pakistan for the treatment of hormonal disorders and oxidative stress. The present study was aimed to evaluate the efficacy of *Launaea procumbens* methanol extract (LPME) against KBrO_3_-induced oxidative stress and hormonal dysfunction in thyroid.

**Methods:**

To examine the effects of LPME against the oxidative stress of KBrO_3_ in thyroid tissue, 36 male albino rats were used. Protective effects of LPME were observed on thyroid hormonal levels, activities of antioxidant enzymes, lipid peroxidation (TBARS) and DNA damage.

**Results:**

Treatment with KBrO_3_ significantly *(P < 0.01)* reduced the levels of T_3_ (55.13 ± 1.93) and T_4_ (14.7 ± 1.78) and increased TSH (55.13 ± 1.93) levels. KBrO_3_ exposure in rats reduced the activities of antioxidant enzymes viz.; CAT (1.16 ± 0.08); SOD (12.0 ± 0.08), GST (17.7 ± 1.1) and GSR (54.3 ± 2.1) but increased lipid peroxidation (20.3 ± 0.71) and DNA (30.4 ± 2.0) damage. Co-administration of LPME significantly (*P < 0.01*) improved these alterations with respect to hormonal levels, activities of antioxidant enzymes and lipid peroxidation close to those seen in control rats.

**Conclusion:**

These results suggest that LPME can protect thyroid tissue against oxidative damage, possibly through the antioxidant effects of its bioactive compounds.

## Background

Potassium bromate (KBrO_3)_ molecular weight 166 g/mol is an oxidizing agent. KBrO_3_ has been used is in industries for the formation of hair solution and cosmetics. Potassium bromate is formed as by product during ozonization of water, causes infections and has been classified as 2B group toxic chemical a probable human carcinogen [9]. KBrO_3_ causes renal cell and thyroid carcinomas in rats, hamsters and mice when exposed chronically [15]. It has been investigated that potassium bromate produces free oxygen radicals which causes oxidative stress and DNA damages [20]. KBrO_3_ causes nephrotoxicity and hepatotoxicity; decreases the tissue soluble proteins, antioxidant enzymes. The decrease of antioxidant enzymes are due to reactive oxygen species (ROS) produced by metabolism of potassium bromate. Potassium bromate depleted GSH content in various tissues which causes decrease in phase II metabolizing enzymes like GSH-Px and glutathione reductase (GSR). It also increases TBARS contents, causes lipid peroxidation and disrupts liver profile including γ-GT, ALP and protein concentration [4]. Medicinal plants and nutraceuticals play important role in improving human health and phytomedicine [24]. These medicinal plants are composed of some bioactive phytochemical substances, regulates various physiological and molecular action in living organisms. Today many natural products extracted from medicinal plants are being tested for the presence of new drugs with new modes of pharmacological action. Special features of higher plants are their capacity to produce a large number of secondary metabolites [2].

*Launaea procumbens* (Roxb.)Amin. (Asteraceae) is an annual herb having simple leaves and yellow flowers found in waste places, vacant lots and in cultivated fields throughout Pakistan. *Launaea procumbens* was used as a food and washing agent [27] rheumatism, galactogogues, and increases milk production. Eye redness and itchiness are treated with *Launaeaprocumbens* and also traditionally used in kidney (painful urination), liver and sexual diseases like gonorrhoea [23]. The antimicrobial activity of ethanolic and aqueous fractions of *Launaeaprocumbens* was checked against various bacterial and fungal pathogens and reported that proved that ethanolic extract show maximum activity while theaqueous extract was inactive. *Launaea procumbens*is generly classified as, Kingdom (Plantae), Order (Asterales), Family (Asteraceae), Tribe (Cichorieae), Genus (*Launaea)*, and Species (*procumbens*). Chemical characterization showed that *Launaeaprocumbens* composed of salicylic acid, vanllic acid, synergic acid, 2-methyl-resercinol, gallic acid and used against plant pathogenic fungi, nematocides and as allelopathic for inhibition of plant growth [26].

In the present research work we arranged to investigate the protective effects of plant extracts against KBrO_3_induced thyroid dysfunctions in rats.

## Methods

### Plant collection and extraction

*Launaea procumbens* whole plant was collected from District Bannu during summer season 2016. After shad drying of plant at room temperature (25 °C), it was ground mechanically to get fine powder. Among powder, 1 kg of fine powder was socked in 70% methanol (1.5 L) and shacked randomly. After a week the extract was filtered viaWhatman filter paper No.1. Filtrate was concentrated using rotary evaporator at 38 °C to get methanol crude extractand was kept at 4 °C in the refrigerator for further in vitro investigation.

### Experimental procedure

Sprague-Dawley male rats were divided into sixteen groups (06 rats). Group 1 was control while Group II received DMSO and saline (0.20% aqueous NaCl solution) orally (Wednesday and Saturday) at a dose of 3 ml/kg b.w. Group III was administered high purity grade KBrO_3_ (99.9%) 20 mg/kg b.w in 0.20% aqueous NaCl solution (Monday and Thursday) intragastrictly. Group IV and V were given 100; 200 mg/kg b.w of crude methanol extract (Wednesday and Saturday) of *L. procumbens* (LPME) after 48 h of KBrO_3_ treatment. However, Group VI was given only (LPME) at a dose of 200 mg/kg b.w (Wednesday and Saturday). All these treatments were given twice a week for 4 weeks. After completion of experiment all the animals were kept on normal feed without any treatment for at least 24 h before the dissection of animals. Animals were given chloroform anesthesia after urine collection and dissected from ventral side. Blood was collected in the falcon tube and centrifuged for serum and stored in refrigerator. Thyroid glandwas removed and washed in ice cold saline, dried with blotting paper and weighted. After weighing the tissues were divided into two portions. One part was cut off and stored in fixative sera for histology while the other portion was treated with liquid N_2_ and stored at −70 °C for further biochemical and molecular studies.

### Serum biochemistry

Serum level of various hormones viz.; T_3_, T_4_and TSHwere calculated through 10,227-Czch Republic (IM1447-IM3286) Kit purchased from IMMUNOTECH Company.

### Antioxidant oxidant profile

Eighty mg tissue of thyroid tissue was homogenized in phosphatebuffer for 20 min at 4 °C and centrifuged at 10,000 rpm to obtain supernatant. Total tissue protein [16] and antioxidant enzymes like CAT and POD [3], SOD [12], γ-GT [6], GSH [1], TBARS [11], GSH-Px, GSR and GST [19, 21, 22], H_2_O_2,_ and QR [10] respectively.

### Genotoxicity assays

DNA damages were assessed using protocol of Wu et al. [28] qualitatively as well as quantitatively.

### Histopathalogical studies

Cellular changes were observed light microscope at 40×.

### Statistical analysis

To determine the treatment effects one way analysis of variance was carried by computer software SPSS 13.0. Level of significance among the various treatments was determined by LSD at 0.05% level of probability.

## Results

### Serum Biochemistry

Effects of methanol fraction on the serum level of thyroid hormones like TSH, T_3_ and T_4_ are shown in Table 1. Treatment of KBrO_3_ caused reduction in the secretion of T_3_, T_4_ and increased the secretion of TSH comparatively to non treated rats. Co-administration of LPME significantly decreased the hormonal secretion in a dose dependent way.

**Table 1:** Serum biochemistry

Treatment	TSH (ng/dl)	T_4_ (ng/ml)	T_3_ (ng/ml)
Control	35.53 ± 1.56++	10.0 ± 2.06++	23.2 ± 1.87++
DMSO + olive oil	34.67 ± 1.75++	9.50 ± 1.04++	22.6 ± 1.94 ++
20 mg/kg KBrO_3_	55.13 ± 1.93^a^	6.0 ± 1.32^a^	14.7 ± 1.78^a^
100 mg/kg LPME+ KBrO_3_	42.07 ± 1.84++	8.5 ± 1.24++	26.43 ± 2.4++
200 mg/kg LPME+ KBrO_3_	36.87 ± 2.02++	9.3 ± 1.06++	24.50 ± 1.75++
200 mg/kg LPME alone	34.0 ± 2.0++	10.7 ± 1.19++	21.67 ± 2.16++

Mean ± SE (*n* = 6 number)

^a^Indicate significance from the control group at *P <* 0.01 probability level

++ Indicate significance from the KBrO_3_ group at *P <* 0.01 probability level

### Assessment of antioxidant profile

Antioxidant enzymes play a crucial role in detoxification of free radicals. KBrO_3_ induction in rats significantly (*p* > 0.01) decreased the amount of tissue soluble protein and activities of first level antioxidant enzymes viz.; CAT, POD and SOD as compare to control. Post-administration of 100 and 200 mg/kg b.w., LPME markedly (*p* > 0.05) reduced the intoxication and reversed the changes to normal level (Table 2).

**Table 2:** Effect of LPME on tissue protein and antioxidant enzymes in thyroid of rat

Treatment	Protein (μg/mg tissue)	CAT (U/min)	POD (U/min)	SOD (U/mg protein)
Control	0.57 ± 0.01++	2.88 ± 0.09++	8.0 ± 0.09++	26.17 ± 0.77++
DMSO + olive oil	0.53 ± 0.01 ++	2.90 ± 0.05++	8.2 ± 0.05++	25.0 ± 0.92++
20 mg/kg KBrO_3_	0.35 ± 0.09^a^	1.16 ± 0.08^a^	6.3 ± 0.20^a^	12.0 ± 0.08^a^
100 mg/kg LPME+ KBrO_3_	0.58 ± 0.03++	2.18 ± 0.04++	7.0 ± 0.29++	18.3 ± 0.60++
200 mg/kg LPME+ KBrO_3_	0.52 ± 0.01++	2.62 ± 0.03++	7.8 ± 0.04++	20.10 ± 1.19++
200 mg/kg LPME alone	0.58 ± 0.01++	2.93 ± 0.09++	8.19 ± 0.08++	28.0 ± 0.82++

Mean ± SE (*n* = 6 number)

^a^Indicate significance from the control group at *P <* 0.01 probability level

++ Indicate significance from the KBrO_3_ group at *P <* 0.01 probability level

Table 3 revealed the activities of phase II metabolizing enzymes viz.; GST, GSH-Px, GSR, QR, and γ-GT in various groups. KBrO_3_-treatment in rats significantly (*p* < 0.01) depleted the activity of GST, GSH-Px, GSR and QR, whereas increased the activity of γ-GT comparatively to control group. The modulation were significantly (*p* > 0.01) reversed by the co-treatment of 100 and 200 mg/kg b.w., LPME near to control group.

**Table 3:** Effect of LPME on thyroid GST, GSR, GSH-Px, γ*-*GT and QR activity in rat

Treatment	GSH-Px (nM/mg protein)	GSR (nM/min/mg protein)	GST(nM/min/mg protein)	γ-GT (nM/min/mg protein)	QR (nM/min/mg protein)
Control	103.3 ± 2.23++	70.7 ± 1.9++	41.7 ± 1.0++	49.3 ± 3.4++	92.7 ± 2.9++
DMSO + olive oil	102.7 ± 1.0++	69.3 ± 2.6++	41.3 ± 0.9++	49.7 ± 3.9++	91.2 ± 2.7++
20 mg/kg KBrO_3_	80.3 ± 2.7^a^	54.3 ± 2.1^a^	17.7 ± 1.1^a^	105.5 ± 1.7^a^	72.5 ± 3.5^a^
100 mg/kg LPME+ KBrO_3_	98.3 ± 1.0++	64.0 ± 2.9++	34.7 ± 1.9++	92.3 ± 2.2++	85.2 ± 3.3++
200 mg/kg LPME+ KBrO_3_	101.0 ± 0.7++	69.0 ± 1.9++	39.5 ± 0.7++	58.5 ± 1.4++	91.4 ± 2.5++
200 mg/kg LPME alone	104.7 ± 2.1++	71.7 ± 1.8++	41.5 ± 0.7++	50.3 ± 2.1++	94.6 ± 2.6++

Mean ± SE (*n* = 6 number)

^a^Indicate significance from the control group at *P <* 0.01 probability level

++ Indicate significance from the KBrO_3_ group at *P <* 0.01 probability level

TBARS, H_2_O_2_, GSH and tissue nitrite are key marker of lipid peroxidation and cellular oxidative stress. KBrO_3_ effects on the content of TBARS, H2O2, and GSH are shown in Table 4. Administration of 20 mg/kg b.w., of KBrO_3_ significantly (*p* < 0.01) reduced the concentrations of GSH while increased H_2_O_2_ and TBARS as compare to control. TBARS, tissue nitrite was significantly reduced (p < 0.01) by LPME decreased the level of TBARS at 100 and 200 mg/kg (*p* < 0.01) as compared to KBrO_3_ group. Level of GSH was improved while H_2_O_2_ was reduced (*p* < 0.01) by LPME at 100 mg/kg and 200 mg/kg b.w, respectively.

**Table 4:** Effect of LPME on thyroid GSH, TBARS, H_2_O_2_

Treatment	TBARS (nM/min/mg protein	GSH (μM/g tissue)	H_2_O_2_ (nM/min/mg tissue)
Control	12.8 ± 1.14++	0.44 ± 0.02++	0.86 ± 0.02++
DMSO + olive oil	13.0 ± 1.06++	0.44 ± 0.03++	0.85 ± 0.02++
20 mg/kg KBrO_3_	20.3 ± 0.71^a^	0.67 ± 0.01^a^	1.03 ± 0.01^a^
100 mg/kg LPME+ KBrO_3_	15.3 ± 1.35++	0.52 ± 0.04++	0.94 ± 0.03++
200 mg/kg LPME+ KBrO_3_	13.3 ± 0.95++	0.44 ± 0.05++	0.88 ± 0.03++
200 mg/kg LPME alone	12.0 ± 0.76++	0.40 ± 0.06++	0.82 ± 0.02++

Mean ± SE (n = 6 number)

^a^Indicate significance from the control group at *P <* 0.01 probability level

+, ++ Indicate significance from the KBrO_3_ group at *P <* 0.01 probability level

### Thyroid weight and genotoxicity

Effects of LPME against KBrO_3_ administration on rats thyroid weight, relative tissue weight and % DNA fragmentation are shown in Table 5. KBrO_3_ caused a significant (*p* < 0.01) increased in thyroid tissue weight and relative thyroid tissue weight and %DNA damages as compared to non treated control group. Post-treatment of LPME erased the toxic effect of KBrO_3_ on rat and reduced the thyroid weight, relative tissue weight and % DNA fragmentation and significantly (*p* < 0.01) reversed towards control group.

**Table 5:** Effect of LPME on thyroid weight, relative thyroid weight and % DNA damages

Treatment	% DNA fragmentation	Thyroid. weight (g)	Relative thyroid weight (% to body weight)
Control	3.8 ± 2.3 ++	55.3 ± 2.7++	0.55 ± 0.02++
DMSO + olive oil	3.9 ± 2.1++	56.5 ± 2.2++	0.56 ± 0.03++
20 mg/kg KBrO_3_	30.4 ± 2.0^a^	79.7 ± 2.9^a^	0.79 ± 0.09^a^
100 mg/kg LPME+ KBrO_3_	7.2 ± 1.8 ++	67.8 ± 2.4++	0.67 ± 0.04++
200 mg/kg LPME+ KBrO_3_	4.4 ± 3.8++	57.3 ± 2.6++	0.57 ± 0.06++
200 mg/kg LPME alone	3.7 ± 2.6++	56.7 ± 2.2++	0.56 ± 0.02++

Mean ± SE (n = 6 number)

^a^Indicate significance from the control group at *P <* 0.01 probability level

++ Indicate significance from the KBrO_3_ group at *P <* 0.01 probability level

### Histopathology of thyroid tissue

Microscopic examinations of various treated groups are shown in Table 6. Administration of KBrO_3_ caused colloid depletion and cellular hypertrophy, blood vessels congestion, inflammatory cells infiltrations and follicular shape disruptionand follicular cells hyperplasia. Post-treatment with LPME significantly erased the injuries near to control rats.

**Table 6:** Effect of LPME on thyroid histopathology

Treatment	Colloids depletion	Hyper-Trophy	Hyperplasia	Blood vessel congestion	Degeneration of follicular architecture
Control	–	–	–	–	–
DMSO + olive oil	–	–	–	–	–
20 mg/kg KBrO_3_	+++	+++	+++	+++	+++
100 mg/kg LPME+ KBrO_3_	–	−/+	–	−+	−/+
200 mg/kg LPME+ KBrO_3_	–	–	–	−+	–
200 mg/kg LPME alone	–	–	–	–	–

-, normal; −/+, mild; ++, medium; +++, severely damaged

## Discussion

Thyroid hormones are essential for normal growth of organs, development, function and also regulate hepatocytes metabolism while liver in turn metabolizes thyroid hormones. It means that liver and thyroid hormones are connected with one another, dysfunction of one causes disturbance of other [17]. The hypothalamus releases thyrotrophic-releasing hormone (TRH), which stimulates pituitary gland to release thyroid stimulating hormone (TSH), which in turn promotes thyroid cells to produce thyroid hormones. When level of thyroid hormone is low than TSH and TRH are high, try to increase thyroid hormone and causes risk of thyroid tumor in rats [5]. Our results showed that administration of KBrO_3_ depleted the secretion of thyroid hormones i.e., T_3_ and T_4_ and elevated TSH level in serum of rats which was recovered by LPME. Similar results were reported by Hamidian et al.*,* [7].

The present study revealed that marked changes were induced by KBrO_3_ in absolute b.w and % increase in body weight. KBrO_3_ treatment of rats significantly decreased the b.w, as compare to the non-treated control group. Our results are similar to the findings reported by Farombi et al. [4] that KBrO_3_ treatment to rat decreases the b.w non significantly as compare to control group. Other study also determined that that low amount of KBrO_3_ is not toxic as well as mutagenic (Yamaguchi et al.*,* [29]. Increased lipid peroxidation and/or altered non-enzymatic and enzymatic antioxidant systems contributed oxidative stress. Various reports revealed that various enzymatic and non-enzymatic systems in mammalian cell have the capability to reduced ROS and other free radicals [25]. Toxicity induced with KBrO_3_ results from its metabolism through CYP 450 into free radicals that in turns leads to oxidative damages. In thyroid presence of cytochrome P450 enzyme system has not been established and injuries induced in thyroid are considered indirectly via the production of metabolites in other organs. In the present study induction of KBrO3 significantly alter the enzymatic level which was reversed by co-treatment of LPME. Similar reports were obtained by the exposure of KBrO_3_ in rats which has been restored by supplementation of *Ficus racemosa* and a natural antioxidant kolaviron [14]. Free radicals react with DNA to form a mutagenic pirimedopurinone adduct (M1G) of deoxyguanosine [18] causes genomic instability and damages. The data revealed that the treatment of KBrO_3_ causes significant oxidative DNA damage in thyroid tissue of rats which are reversed by LPME. Similar investigation was reported by Khan et al.*,* [13] during study of protective effects of *Digeramuricata*. These results show that the extract contains bioactive phenolic and poly phenolic compounds which play important role in DNA repair. Data of the present study revealed that KBrO_3_ treatment in rats caused colloids depletion and hypertrophy, blood vessels congestion, follicular shape degradation and hyperplasia in thyroid. Treatment with LPME significantly erased the injuries. Similar observation was reported by Hooth et al. [8] during drinking water exposure of sodium chlorate in rats.

## Abbreviations

GSR: Glutathione reductase
KBrO_3_: Potassium bromate
ROS: Reactive oxygen species
TRH: Thyrotrophic-releasing hormone
TSH: Thyroid stimulating hormone
